# Immunodiagnosis of *Fasciola gigantica* Infection Using Monoclonal Antibody-Based Sandwich ELISA and Immunochromatographic Assay for Detection of Circulating Cathepsin L1 Protease

**DOI:** 10.1371/journal.pone.0145650

**Published:** 2016-01-05

**Authors:** Panat Anuracpreeda, Runglawan Chawengkirttikul, Prasert Sobhon

**Affiliations:** 1 Division of Agricultural Science, Mahidol University, Kanchanaburi Campus, Saiyok, Kanchanaburi, Thailand; 2 Department of Anatomy, Faculty of Science, Mahidol University, Rama VI Road, Bangkok, Thailand; 3 Department of Microbiology, Faculty of Science, Mahidol University, Rama VI Road, Bangkok, Thailand; Second University of Naples, ITALY

## Abstract

**Background:**

Tropical fasciolosis caused by *Fasciola gigantica* infection is one of the major diseases infecting ruminants in the tropical regions of Africa and Asia including Thailand. Parasitological diagnosis of fasciolosis is often unreliable and possesses low sensitivity. Therefore, the detection of circulating parasite antigens is thought to be a better alternative for diagnosis of fasciolosis, as it reflects the real parasite burden.

**Methods:**

In this study, we have produced a monoclonal antibody (MoAb) against recombinant *F*. *gigantica* cathepsin L1 (rFgCatL1), and developed both sandwich enzyme-linked immunosorbent assay (sandwich ELISA) and immunochromatographic (IC) test for rapid detection of circulating cathepsin L1 protease (CatL1) in the sera from mice experimentally and cattle naturally infected with *Fasciola gigantica*. MoAb 4E3 and biotinylated rabbit anti-recombinant CatL1 antibody were selected due to their high reactivities and specificities.

**Results:**

The lower detection limits of sandwich ELISA and IC test were 3 pg/ml and 0.256 ng/ml, respectively. Sandwich ELISA and IC test could detect *F*. *gigantica* infection from day 1 to 35 post infection. In experimental mice, the sensitivity, specificity and accuracy were 95%, 100% and 98.6% (for sandwich ELISA), and 93%, 100% and 98.2% (for IC test), while in natural cattle they were 98.3%, 100% and 99.5% (for sandwich ELISA), and 96.7%, 100% and 99.1% (for IC test).

**Conclusions:**

These two assay methods showed high efficiencies and precisions for diagnosis of fasciolosis by *F*. *gigantica*.

## Introduction

Tropical fasciolosis caused by *Fasciola gigantica* infection is one of the major diseases infecting ruminants in the tropical regions of Asia and Africa, and causes a significant economic loss in livestock industry in developing and underdeveloped countries for more than 3.2 billion US dollars per annum [1]. In Thailand, the prevalent rate of fasciolosis in cattle and buffaloes is estimated to be 4–24%, with the highest incidents in the Northeast (up to 85% of cattle and buffaloes) [2, 3]. For Thailand alone, the annual economic loss due to fasciolosis in cattle and buffaloes is estimated to be at least 350 million bahts (US $10 million) [2]. Moreover, liver fluke infection in humans is also recognized by the World Health Organization as a major public health problem, and recent reports estimate that at least 2.4 million, up to 17 million people are presently infected worldwide and about 91 million are at risk [4]. Presently, the diagnosis of fasciolosis is based on the microscopic identification of the fluke’s eggs in the feces. However, during prepatent period of the disease (13 to 14 weeks after infection), the flukes do not produce eggs, and thus the eggs in feces cannot be detected [5]. The serodiagnostic method for the detection of antibody in animals has been developed for the diagnosis of fasciolosis [5–7]; however, the circulating antibodies appear in the serum for several months after the infection even though the parasites may have already been killed. Therefore, the detection of circulating parasite antigens is thought to be a better alternative for diagnosis of fasciolosis, as it reflects the real parasite burden. Only a few detections of circulating antigens in animals with fasciolosis have been reported with variable efficiencies due largely to their availabilities in the circulation [8–13].

Cathepsin L proteinases (CatLs) are one group of endopeptidases which is expressed at a high level in *Fasciola* flukes. CatLs are secreted in copious amounts by gastrodermal epithelial cells of immature and adult *Fasciola* sp. [14], thus should provide a highly sensitive target for diagnosis. In *F*. *gigantica*, several isotypes of CatLs are isolated from the excretory-secretory (ES) products of adult flukes. These enzymes include FgCatL1 [15], FgCatL2 [16], and FgCatL3 [17] which exhibit molecular weight at 27 kDa, 27–28 kDa, and 28 kDa, respectively. In addition, several CatL cDNAs and recombinant proteins have been cloned and expressed from adult flukes, namely rFgCL1 [18], rFgCL1-D [19], and rFgCL2 [20]. CatLs are synthesized and packaged in vesicles within the caecal epithelial cells of *Fasciola* sp. as other digestive enzymes and are released into the host fluid in a fairly large amount [21]. In this study, we have produced a monoclonal antibody (MoAb) against recombinant *F*. *gigantica* cathepsin L1 (rFgCatL1), and developed both sandwich enzyme-linked immunosorbent assay (sandwich ELISA) and immunochromatographic (IC) test for rapid detection of circulating CatL1 antigen of *F*. *gigantica* in the sera of experimentally and naturally infected animals. These immunodiagnostic assays showed high sensitivities, specificities and accuracies for fasciolosis by *F*. *gigantica* in ruminants [12–13, 22].

## Materials and Methods

### Collection of parasite specimens

*F*. *gigantica* metacercariae were obtained from experimentally snails, *Lymnaea ollula*, which were infected with *F*. *gigantica* miracidiae. After 6–8 weeks, the cercariae were shed from the snails and settled on the 5 x 5 cm cellophane papers and developed into metacercariae. The metacercariae were collected from cellophane papers and washed several times with Hank’s balance salt (HBS) solution containing 100 U/ml penicillin and 100 mg/ml streptomycin and used immediately [13, 22].

A method described by Anuracpreeda et al. [13, 22] was used to obtain newly excysted juveniles (NEJ) of *F*. *gigantica*. Briefly, the metacercariae (Met) were excysted by incubation in distilled water containing 1% (w/v) pepsin (pepsin A from porcine gastric mucosa, P-7000, Sigma–Aldrich Co.) and 0.4% (v/v) HCl at 37°C for 45 min. After washing with distilled water, they were resuspended in a solution containing 0.02 M sodium dithionite (Fluka Biochemika), 0.2% (w/v) taurocholic acid (T-4009, Sigma–Aldrich Co.), 1% (w/v) NaHCO_3_ and 0.8% (w/v) NaCl. As well, 0.005% (v/v) HCl was added to the tube to generate CO_2_ gas. Subsequently, parasite specimens were incubated at 37°C for 45 min and washed with distilled water. The activated metacercariae were transferred to fresh RPMI-1640 medium (Sigma Chemical Co., St. Louis, MO, USA) containing 10 μg/ml gentamycin, and 10% fetal bovine serum. Then, the contents were transferred to an excystment tower fitted with 100 μm meshes placed within a 24-well plate and incubated at 37°C overnight. On the following day, the NEJ were collected and washed several times with Hank’s balance salt (HBS) solution and used immediately [13, 22].

To obtain juveniles of *F*. *gigantica*, male Golden Syrian hamsters were orally infected with 30 metacercariae per each animal. At 1, 3, and 5 weeks after infections, the juvenile parasites were collected from the infected animals by sacrificing and teasing the liver to collect the parasites. The worms were washed several times with Hank’s balance salt (HBS) solution and used immediately [22].

Adult stages of *F*. *gigantica* and *F*. *hepatica* were obtained from the intrahepatic bile ducts and gall bladders of naturally infected cattle or water buffaloes killed at the local abattoirs. Other parasites collected from the same group of animals for the cross-reactivity study included trematodes (*Gigantocotyle explanatum*, *Eurytrema pancreaticum*, *Paramphistomum cervi*, *P*. *gracile*, *Cotylophoron cotylophorum*, *Fischoederius cobboldi*, *Gastrothylax crumenifer*, *and Schistosoma spindale*), cestodes (*Moniezia benedeni and Avitellina centripunctata*), and nematode parasites (*Trichuris* sp., *Haemonchus placei* and *Setaria labiato-papillosa*). Adult *Schistosoma* sp. (*S*. *mansoni*, *S*.*mekongi* and *S*. *japonicum*) were obtained by perfusing mice 8 weeks after being infected with schistosome cercariae. Adult *Opisthorchis viverrini* were collected from adult golden Syrian hamsters infected with metacercariae obtained from the muscles of naturally-infected cyprinoid fishes. After 4 weeks of infection, the adult *worms* were collected by teasing the liver apart. All parasite samples were washed several times with Hank’s balance salt (HBS) solution before being processed for further experiments [23].

### Preparations of antigens

Crude extract (CE) of the parasites were carried out according to the method described by Anuracpreeda et al. [12–13, 22]. The WB antigens (metacercariae, NEJ, 1, 3, 5-week-old juveniles, adults of *F*. *gigantica* and other species) were obtained by extracting whole *F*. *gigantica* and other parasites in lysis buffer containing 10 mM Tris-HCl, pH 7.2, 150 mM NaCl, 0.5% (v/v) Triton X-100, 1 mM EDTA and 1 mM PMSF (P-7626, Sigma-Aldrich Co.). The parasite samples were homogenized, and then sonicated for 5–10 min with 5-s pulse and pause cycles in an ice bath. Thereafter, the suspensions were rotated at 4°C for 1 h and centrifuged at 5,000 x g at 4°C for 20 min to get rid of the eggs. Finally, the supernatants were collected, lyophilized, and stored at −70°C until use in later experiments.

The method described by Anuracpreeda et al. [12, 22] was used to obtain ES antigens. Briefly, ES antigens were prepared by incubating freshly collected, live adult flukes in RPMI-1640 media for 3 h at 37°C. After incubation, the parasites’ eggs in the culture medium were discarded by centrifugation at 5000 x g at 4°C for 20 min. The supernatant was collected and dialyzed in 0.01 M PBS, pH 7.2, at 4°C for 24 h, using Spectra/Por dialysis membrane before it was filtered, lyophilized and stored at −70°C until use in later experiments.

Protein concentrations in the parasites’ extracts including CE and ES were estimated by the method of Lowry [24] using bovine serum albumin as a standard. These extracts were stored at -70°C until use.

### Preparation of recombinant *F*. *gigantica* cathepsin L1 (rFgCatL1)

The rFgCatL1 was obatined according to the methods described by Grams et al. [18] and Meemon et al. [25]. Briefly, a fragment of FgCatL1 of adult *F*. *gigantica* was isolated and subcloned into pGEM^®^-T Easy vector (Promega, Madison, USA). The sequence was confirmed by DNA sequence analysis (Macrogen, South Korea). The full-length cDNA of FgCatL1 was subcloned into the bacterial expression vector, pET-30b (Novagen), which was transformed into *Escherichia coli* BL21 (DE). The rFgCatL1 was expressed at 37°C by inducing the bacteria with isopropyl-β-D-thiogalactoside (IPTG), and purified by nickel-nitrilotriacetic acid (Ni^2+^-NTA) affinity chromatography (QIAGEN) at room temperature. Thereafter, the column was washed twice with a washing buffer (50 mM NaH_2_PO_4_, 300 mM NaCl, 20 mM imidazole, pH 8.0), and the protein was eluted by an elution buffer (50 mM NaH_2_PO_4_, 300 mM NaCl, 250 mM imidazole, pH 8.0). The eluate was dialyzed against 0.01 M PBS, pH 7.4, at 4°C overnight, and concentrated by membrane filtration using an Amicon Ultra centrifugal filter devices 10,000 NMWL (Millipore, Bedford, MA, USA), and dissolved in 0.01 M PBS, pH 7.4. Purifed rFgCatL1 was stored at -70°C until used in further experiments.

### Production of monoclonal antibodies (MoAbs) against rFgCatL1

Inbred eight week-old female BALB/c mice were immunized with rFgCatL1 according to Anuracpreeda et al. [22]. The hybridoma clones expressing MoAb against rFgCatL1 were produced by fusion of the splenocytes from immunized BALB/c mouse with non-secreting mouse myeloma cells (P3x63-Ag8.653), using polyethylene glycol (PEG) (Sigma-Aldrich Inc., St. Louis, MO, USA). The MoAbs produced by the hybridoma cells were screened by indirect enzyme-linked immunosorbent assay (Indirect ELISA), and the highly reactive hybridomas were cloned by limiting dilution methods using a feeder layer of splenocytes. One hybridoma clone (4E3) producing a high titer of antibody against rFgCatL1, as screened by indirect ELISA, was selected. The specific MoAb class and subclass were done by ELISA using the SBA Clonotyping™ System/HRP (SouthernBiotech, Birmingham, USA).

### Assay for assessing the specificity of MoAb 4E3

Indirect ELISA was used for examining the specificity of MoAb 4E3 according to the method of Anuracpreeda et al. [22]. Briefly, ninety six-well microtiter plate (Nunc A/S, Roskilde, Denmark) was coated with 50 μl of 10 μg/ml of CE from *F*. *gigantica* as well as CE from other trematode, cestode and nematode parasites diluted in coating buffer (15 mM Na_2_CO_3_, 35 mM NaHCO_3_, pH 9.6), and incubated at 37°C overnight. The coated plate was washed three times with distilled water, and each time the washing fluid was left in the wells for approximately 1 min. Subsequently, the plate was blocked with 50 μl/well of blocking solution (0.25% bovine serum albumin (BSA), 0.05% Tween 20) (Sigma) in 0.01 M PBS, pH 7.2, at 37°C for 30 min. Thereafter, the plate was similarly washed, and 50 μl of undiluted and diluted MoAb 4E3 at 1:10, 1:100, 1:500 and 1:1,000 were added, and incubated at 37°C for 2 h. After washing, the plate was incubated with 50 μl/well of horseradish peroxidase (HRP)-conjugated goat anti-mouse immunoglobulin (Sigma-Aldrich Inc.) at 1:6000 dilution in the blocking solution at 37°C for 1 h. Then the plate was washed with distilled water as previously described, the color development was generated by adding 50 μl/well of 3,3',5,5'-tetramethyl benzidine (TMB) substrate (KPL, Gaithersburg, USA). The enzymatic reaction was allowed to take place at room temperature for 10 min. Finally, the enzymatic reaction was stopped by the addition of 50 μl 1N HCl. The optical density (OD) value at 450 nm was measured using a microplate reader (Multiskan Ascent, Labsystems, Helsinki, Finland).

In the cross-reactivity study, proteins in CE from *F*. *gigantica*, as well as from other trematode, cestode and nematode parasites, were separated by 12.5% Sodium dodecyl sulphate-polyacrylamide gel electrophoresis (SDS-PAGE) according to Laemmli [26], and then electrophoretically transferred onto nitrocellulose membranes (Bio-Rad, Philadelphia, PA, USA) for immunoblotting [27]. The non specific binding was blocked with 5% skimmed milk in Tris buffered saline (TBS) pH 7.4 containing 0.05% Tween 20 at room temperature for 2 h. Subsequently, the membranes were incubated in undiluted hybridoma fluid (MoAb 4E3) and myeloma culture fluid (as the negative control) at room temperature for 2 h. Thereafter, the membranes were washed with TBS, and incubated with the HRP-conjugated goat anti-mouse immunoglobulin (Sigma-Aldrich Inc.), diluted to 1:4000 with 1% skimmed milk in TBS pH 7.4, containing 0.05% Tween 20 at room temperature for 1 h. After washing as previously described, the color was developed in the dark using 3,3',5,5'-tetramethyl benzidine (TMB) substrate (KPL, Gaithersburg, USA) at room temperature for 5 min until the positive bands appeared. Finally, the reaction was stopped by adding distilled water.

The method of indirect immunofluorescence detection described by Anuracpreeda et al. [28] was used to detect CatL1 antigen in adult *F*. *gigantica*. Briefly, freshly collected parasites were fixed in 4% (w/v) paraformaldehyde in 0.1 M PBS pH 7.4 at 4°C for 4 h. Then the specimens were immersed in 5% and 30% (w/v) sucrose in 0.1 M PBS at 4°C for 24 h and for 1–2 weeks, respectively. The specimens were embedded in the Tissue Tek O.C.T. medium (Miles Inc., Elkhart, USA) and frozen at −20°C. They were cryo-sectioned at the thickness about 5–7 μm using Leica CM 1800 cryostat, and mounted onto the 3-aminopropyltriethoxy-saline (APES) (Sigma–Aldrich Co.) coated slides. Frozen sections were fixed in acetone at −10°C for 10 min. Thereafter, the sections were washed with 0.01 M PBS, pH 7.4, for 5 min, and incubated in 0.1% glycine in 0.01 M PBS for 15 min, and subsequently in 4% BSA in 0.01 M PBS for 30 min. Then, the sections were incubated with specific MoAb 4E3 at room temperature for 1 h. After washing three times with 0.01 M PBS, pH 7.4, for 5 min each, the sections were treated with fluorescein isothiocyanate (FITC)-conjugated goat anti-mouse IgG (Sigma–Aldrich Co.), diluted to 1:100 with PBS containing 0.05% Tween-20 at room temperature for 30 min. Finally, the treated sections were rinsed thoroughly with PBS, covered with Vecta Shield H-1000 (Vector Laboratories Inc., Burlingame, CA, USA), observeded and photographed under a EVOS FL color fluorescence microscope.

### Production of polyclonal antibody (PoAb) against rFgCatL1

Polyclonal anti-rFgCatL1 was produced by immunizing New Zealand White rabbits with rFgCatL1 according to the method described by Anuracpreeda et al. [13]. Briefly, the animals were immunnized by subcutaneous injection with 500 μg of rFgCatL1 mixed with Freund’s adjuvant (Sigma-Aldrich Inc.) three times at 3-week intervals, using Freund’s complete adjuvant in the prime injection and incomplete adjuvant in the first and second boosts via the same route. The rabbits were bled, and the antibody titers in the antisera were tested by indirect ELISA against rFgCatL1.

### Purification of MoAb 4E3 and PoAb against rFgCatL1

For sandwich ELISA and immunochromatographic (IC) test, the IgG fraction of both MoAb and PoAb was purified by ammonium sulfate precipitation, dialyzed against an excess of PBS and applied to an affinity chromatography in a Mab trap protein G Sepharose column (Amersham Pharmacia Biotech AB, Uppsala, Sweden). For sandwich ELISA, the purified IgG of PoAb was conjugated with biotin using N-hydroxysuccinimidobiotin (Sigma Co) as previously described by Anuracpreeda et al. [13].

### Animal infection

A total of 120 male 5-week-old ICR mice from National Laboratory Animal Center, Mahidol University, Nakorn Pathom, Thailand, were randomly divided into two groups: non-infected control and infected groups, sixty control and sixty infected mice were assigned to 5 subgroups of 12 mice per group. They were killed at day 1, 4, 7, 21 and 35 post infection. The treated groups were orally infected with 30 viable *F*. *gigantica* metacercariae per mouse, whereas control groups received 0.5 ml of 0.85% NaCl solution. At necropsy time, whole blood samples of all animals were collected aseptically into tubes without anticoagulant, and they were separated by centrifugation for collection of the sera. For cross-reactivity study, twenty sera were collected from mice infected with *O*. *viverrini* and 20 sera from hamsters infected with *S*. *mansoni*. As well, sixty sera from non-infected hamsters were collected and used as the negative control.

### Sera from naturally infected cattle

Sera were collected from fields in many areas of Thailand with monoinfections; these include 60 serum samples from cattle infected with *F*. *gigantica* (fasciolosis), 50 samples from *P*. *cervi* (paramphistomosis), 10 samples from *M*. *benedeni* (monieziasis), 10 samples from *Strongyloides* sp. (strongyloidiasis), 10 samples from strongylids (strongylid infection), and 10 samples from *Trichuris* sp. (trichuriasis). Sixty non-infected cattle sera were obtained and used for the negative control. All faecal samples which were collected from the rectum of the animals were diagnosed for *Fasciola* eggs by sedimentation method [29] and for other parasite eggs by flotation method [30].

### The lower detection limit and the specificity of sandwich ELISA

The cut-off point was calculated as the mean optical density (OD) of negative controls plus three standard deviations (SD). The OD readings equal to or less than the cut-off point were considered negative while those readings greater than the cut-off point were considered positive. The lower detection limit of sandwich ELISA was estimated by titration with a serial dilutions of *F*. *gigantica* rCatL1 and CE antigens of metacercariae, NEJ, 1, 3, 5-week-old juveniles, adults and ES antigen of adult, from 0.04 pg/ml to 20 μg/ml. The end point of detection limit was considered to be the lowest amount of antigen still giving the positive OD values. To determine cross-reactivities studies, the specificity of ELISA assay was analysed by using CE from other trematode, nematode and cestode parasites. Each of these antigens was prepared at various concentrations and detected possible presence of CatL1 antigen.

### Detection of circulating *F*. *gigantica* CatL1 antigen by sandwich ELISA

The sandwich ELISA for detection of circulating antigen followed the method described by Anuracpreeda et al. [13]. For each step, 50 μl/well was added unless otherwise mentioned. Ninety six-well plate (Nunc A/S, Roskilde, Denmark) was sensitized overnight at 4°C with rabbit anti mouse IgG (Dako A/S, Glostrup, Denmark) (10 μg/ml of 0.05 M carbonate buffer, pH 9.6). The coated plate was throughly washed once with 0.05% Tween 20 in normal saline solution (NSST) to remove excess antibody. Each time the washing fluid was left in the wells for approximately 1 min at room temperature. The purified MoAb 4E3 diluted in 1% BSA in PBS pH 7.2 (10 μg/ml) was added and incubated for 3 h at 37°C. After washing 3 times with the same washing buffer, unbound sites were blocked with 150 μl/well of 5% skim milk in PBS for 1 h at 37°C. Thereafter, the plate was washed 3 times, incubated with reference antigens or samples (triplicate wells) for overnight at 4°C. After washing, biotinylated rabbit IgG antibody against rFgCatL1 (2 μg/ml of 1% BSA-0.05% PBST) was added. Then the plate was incubated for 90 min at 37°C and washed with the same washing buffer. Thereafter, streptavidin-conjugated peroxidase (Zymed Laboratory Inc.) at the dilution 1:6000 in 1% BSA-0.05% PBST was added to each well. Finally, TMB substrate solution (KPL, Gaithersburg, USA) was added and incubated in a dark room for 10 min at room temperature, and then the color reaction was monitored. The reaction was stopped by adding of 1 N HCl. The OD values were read in a microplate reader (Multiskan Ascent, Labsystems, Helsinki, Finland) at 450 nm.

### Antibody detection by indirect ELISA

The protocol of indirect ELISA used for detection of the antibody against CatL1 in the sera of infected animals was followed as per the method described by Anuracpreeda et al. [22]. The rFgCatL1 (1 μg/ml) was used for coating a microtiter plate, and allowed to react with the sera of non-infected or infected mice. The HRP–conjugated goat anti-mouse antibody was placed in each well. After substrate addition, the reaction was stopped after 10 min and read at 450 nm.

### Preparation of immunochromatographic (IC) strip test

The immunochromatographic (IC) strip comprised of a sample pad, a conjugate pad, an immobilized nitrocellulose membrane and an absorbent pad.

#### Preparation of colloidal gold probe

The 25 nm colloidal gold (Sigma-Aldrich Inc.) was used for conjugation of IgG. The pH of the colloidal gold solution was adjusted to 9.0 with 0.2 M Na_2_CO_3_. One milliliter of colloidal gold was mixed with 50 μl of purified IgG of PoAb (0.1 mg/ml), and shaken for 30 min at room temperature. Then, 10% BSA solution in 20 mM Na_2_B_4_O_7_ (pH 9.0) was added, and the mixture was incubated for 10 min at room temperature. Thereafter, the conjugate was centrifuged at 12,000 x g for 15 min at room temperature and the supernatant was discarded. The pellet was re-suspended with washing buffer (1% BSA in 20 mM Na_2_B_4_O_7_, pH 9.0), and the coated gold particles were centrifuged. Finally, the precipitate was resuspened in 1% BSA dissolved in 20 mM Tris/HCl buffer, pH 8.2 and store at 4°C for use.

#### Preparation of the conjugate pad

The glass fibers (Sigma-Aldrich Inc.) were used for conjugate pad. They were cut into a size of 5×150 mm and saturated with the colloidal gold probe. The impregnated conjugate pads were dried for 1 h at 37°C and stored in a desicator at room temperature.

#### Immobilization of antibody on nitrocellulose membrane

After being pre-incubated in a humidity control cabinet for 30 min at 25% relative humidity, the nitrocellulose membrane (15×150 mm, Millipore, MA, USA) was immediately coated with 0.5 mg/ml purified MoAb 4E3 (Test antibody) and 0.5 mg/ml goat anti-rabbit IgG antibody (Control antibody). Then, the coated membrane was immediately placed in the humidified box and incubated for 30 min at room temperature. Thereafter, the immobilized membranes were dried in the humidity control cabinet under identical settings. Subsequently, the remaining active sites on the surface of the membrane were blocked by incubation with blocking reagent (0.5% BSA in 10 mM Tris/HCl, pH 7.4) for 10 min at room temperature. The membrane was soaked to 5% sucrose solution for 1 min at room temperature. After drying in the cabinet for 30 min, the membrane was sealed and stored under dried condition in a desicator at room temperature

#### Preparation of sample and absorbent pads

The sample and absorbent pads (Millipore, MA, USA) were cut into a size of 20×150 mm. The sample pad was dipped with a buffer solution containing 2% Triton X-100 in 0.05 M Tris/HCl, pH 7.5, 0.05% polyvinylpyrrolidone for 20 min at room temperature, then dried and stored in a desicator at room temperature.

#### Assembling of IC strip

The sample pad, conjugate pad, immobilized membrane, and absorbent pad were manually overlapped and attached permanently to a plastic backing plate and then cut into 3 mm width strips. These strips were sealed in plastic bags containing desiccating gel and stored at 4°C until required.

### Determination of the sensitivity and specificity of IC strip test

To determine the sensitivity of IC strip, the final optimized IC test was carried out on a 96-well microtiter plate. Various concentrations (20,000 to 0.01 ng/ml in PBS) of rCatL1, CE and ES antigens of adult *F*. *gigantica* were prepared and tested. The lowest concentration of these antigens at which a positive reading was observed represented the sensitivity of the IC test. The specificity of IC strip was studied using antigens from other trematode, cestode and nematode parasites at various concentrations.

### Determination of the stability of IC strip test

To evaluate the stability of the IC strips, they were prepared and stored at room temperature in sealed plastic bags containing desiccating gel. Every week, the strips were taken out for testing both positive and negative sera samples. The test results were recorded and compared.

### Detection of circulating CatL1 of *F*. *gigantica* by the IC strip test

The samples were applied to the sample pad and allowed to react with the colloidal gold-purified anti-rFgCatL1 PoAb conjugate. The mixture flowed through the nitrocellulose membrane chromatographically by the capillary action. After 3–5 min, a positive test showed two red dots for the test and control regions, while a negative test only exhibited one red dot in the control region. The strip test was invalid when there was no red dot at the control region.

The serum samples from both experimentally and naturally infected animals were diluted 1:10 with PBS and placed in a well of 96-well plate. Then, the strips were dipped into the sample solution and taken out for 3–5 min to observe the result. If both the test and control regions showed red dot, the samples were recorded as positive indicating the presence of CatL1 antigen. On the contrary, if the control region showed red dot but not in the test region, the sample is considered as negative.

### Statistical analysis

Each serum sample was tested in duplicate and expressed as an individual mean OD. All data from the detection of CatL1 in sera of experimentally mice and naturally cattle infected with *F*. *gigantica* were calculated. Independent-sample t-test using SPSS computer program (version 19.0 Windows) (SPSS Inc., Chicago, Illinois) was used for data analysis. The *p*-value more than 0.05 was considered not significant and less than 0.05 and 0.01 was considered to be highly and very highly significant, respectively. Standard diagnostic indices including sensitivity, specificity, predictive values, false positive and negative rate, and accuracy were calculated using the method of Galen [31]. These values were calculated and expressed as follows: sensitivity = [no. of true positives / (no. of true positives + no. of false negatives)] x 100, specificity = [no. of true negatives / (no. of true negatives + no. of false positives)] x 100, positive predictive value = [no. of true positives / (no. of true positives + no. of false positives)] x 100, negative predictive value = [no. of true negatives / (no. of true negatives + no. of false negatives)] x 100, accuracy = [all with true positives and negatives / all test done] x 100. The primary data of the sandwich ELISA and IC test are as follows: true negative = number of control samples (other parasitosis and non-infected controls) that show negative result, true positive = number of proven *F*. *gigantica* infection samples that show positive result, false positive = number of control samples that show positive result, false negative = number of proven *F*. *gigantica* infection samples that show negative result.

### Ethics statement

Experiments on all animals were approved by the Animal Care and Use Committee (SCMUACUC), Faculty of Science, Mahidol University, Thailand (Protocol No. MUSC56-013-275), and were specifically used for this study. National Laboratory Animal Center, Nakorn Pathom, Mahidol University, Thailand has earned AAALAC international accreditation. At necropsy day, all animals were anaesthetized with sodium pentobarbital overdose by intra-peritoneal injection (for mice and hamsters) and intravenous injection (for rabbits). The respiratory rate, toe pinch, and pedal reflex of the animals were assessed to ensure general anesthetic condition. The thoraco-abdominal cavity of mice and hamsters was opened, while the cardiac withdrawal was performed to collect all blood from the rabbits. The heart sound of animals was assessed to ensure euthanasia. We received consent to collect the parasite specimens from animals at the abattoir. The cattle sera were obtained from Department of Livestock, Ministry of Agriculture and Co-operatives, Bangkok, Thailand.

## Results

### Preparation of recombinant *F*. *gigantica* cathepsin L1 protease (rFgCatL1)

We previously demonstrated that the full-length FgCatL1 cDNA was successfully subcloned into the bacterial expression vector, pET-30b (Novagen), which was transformed into *Escherichia coli* BL21 (DE). The recombinant FgCatL1 (rFgCatL1) was purified and eluted from by Ni^2+^-NTA column (QIAGEN) and size-separated by SDS-PAGE giving a molecular weight of approximately 30 kDa.

### Production and screening of monoclonal antibodies (MoAbs) against rFgCatL1

In our previous study, we have used the purified rFgCatL1 to immunize the BALB/c mice for production of the specific MoAbs. It was found that culture fluid from seven hybridoma clones (1E10, 1F5, 3D11, 4B10, 4D3, 4E3 and 5E7) exhibited specific binding to rFgCatL1 of *F*. *gigantica*. The immunoglobulin isotypes of the MoAbs from clones 1E10, 1F5 and 3D11 were found to be IgM, while from clones 4B10, 4D3, 4E3 and 5E7 were IgG_1_. All MoAbs were κ light chain isotype. Moreover, MoAb 4E3 showed the highest OD values of antibody reactivities against rFgCatL1 and the native FgCatL1 in CE and ES fractions when screened by indirect ELISA; therefore, it has been used in the present study.

### The specificity of MoAb 4E3

**Fig 1:**
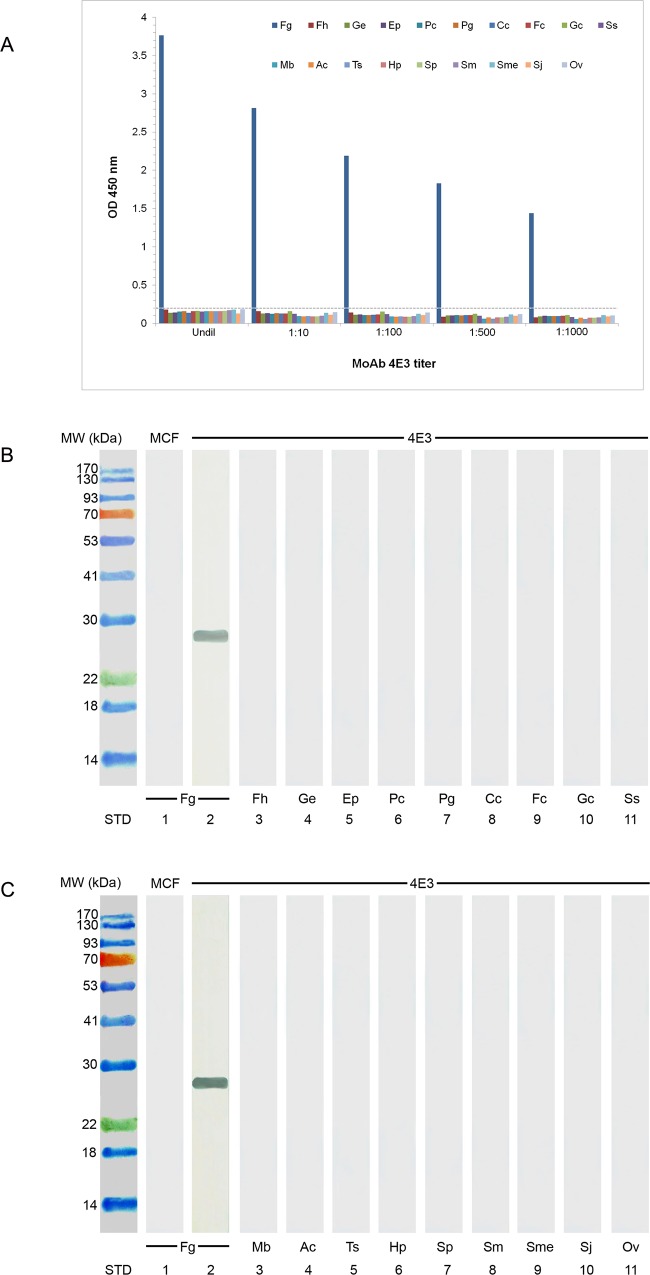
Determination of the specificity of MoAb 4E3 with CE from *F*. *gigantica*, other trematode, cestode and nematode parasites by using indirect ELISA and immunoblot analysis. (A) ELISA OD values of cross-reactivities of MoAb 4E3 and antigens from various trematode, cestode and nematode species. Fg = *F*. *gigantica*, Fh = *F*. *hepatica*, Ge = *G*. *explanatum*, Ep = *E*. *pancreaticum*, Pc = *P*. *cervi*, Pg = *P*. *gracile*, Cc = *C*. *cotylophorum*, Fc = *F*. *cobboldi*, Gc = *G*. *crumenifer*, Ss = *S*. *spindale*, Mb = *M*. *benedeni*, Ac = *A*. *centripunctata*, Ts = *Trichuris* sp., Hp = *H*. *placei* and Sp = *S*. *labiato-papillosa*, Sm = *S*. *mansoni*, Sme = *S*. *mekongi*, Sj = *S*. *japonicum*, and Ov = *O*. *viverrini*. Undil = undiluted MoAb hybridoma fluid, MoAb dilution = MoAb hybridoma fluid diluted at 1:10, 1: 100, 1: 500 and 1: 1,000. The cut-off value is indicated by a horizontal dashed line and calculated as the mean OD of negative controls plus 3 SD. The OD values greater than this cut-off value are considered to be positive. (B) Immunoblotting detection of the cross-reactivities of MoAb 4E3 with CE from *F*. *gigantica* and other trematode parasites. Fg = *F*. *gigantica* (lane 2), Fh = *F*. *hepatica* (lane 3), Ge = *G*. *explanatum* (lane 4), Ep = *E*. *pancreaticum* (lane 5), Pc = *P*. *cervi* (lane 6), Pg = *P*. *gracile* (lane 7), Cc = *C*. *cotylophorum* (lane 8), Fc = *F*. *cobboldi* (lane 9) and Gc = *G*. *crumenifer* (lane 10) and Ss = *S*. *spindale* (lane 11), (C) Immunoblotting detection of the cross-reactivities of MoAb 4E3 with CE from *F*. *gigantica*, other trematode, cestode and nematode parasites. Mb = *M*. *benedeni* (lane 3), Ac = *A*. *centripunctata* (lane 4), Ts = *Trichuris* sp. (lane 5), Hp = *H*. *placei* (lane 6), Sp = *S*. *labiato-papillosa* (lane 7), Sm = *S*. *mansoni* (lane 8), Sme = *S*. *mekongi* (lane 9), Sj = *S*. *japonicum* (lane 10) and Ov = *O*. *viverrini* (lane 11). Lane 1 of B and C is CE from *F*. *gigantica* blotted with the myeloma culture fluid (MCF), which is used as the negative control. STD is the lane containing standard protein molecular weight markers indicated on the left side.

Indirect ELISA (Fig 1A) and immunoblotting assay (Fig 1B and 1C) were used for studying the specficity of MoAb 4E3. It was found that this MoAb 4E3 exhibited strong reaction with the native CatL1 antigen in CE of adult *F*. *gigantica* at molecular weight 27 kDa, while showing no cross-reactivity with CE from other parasites, e.g., *F*. *hepatica*, *G*. *explanatum*, *E*. *pancreaticum*, *P*. *cervi*, *P*. *gracile*, *C*. *cotylophorum*, *F*. *cobboldi*, *G*. *crumenifer*, *S*. *spindale*, *M*. *benedeni*, *A*. *centripunctata*, *Trichuris sp*, *H*. *placei*, *S*. *labiato-papillosa*, *S*. *mansoni*, *S*. *mekongi*, *S*. *japonicum and O*. *viverrini*.

**Fig 2:**
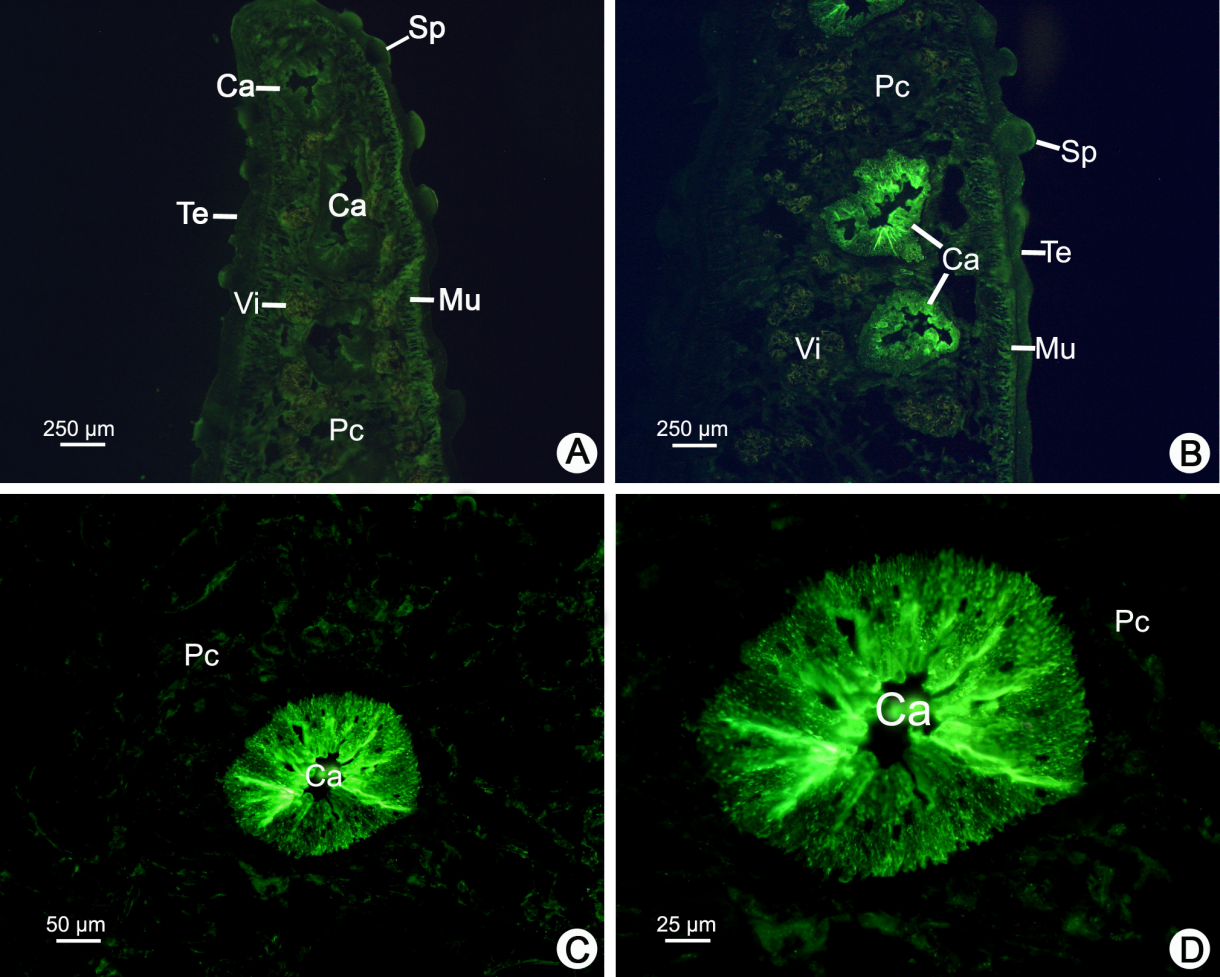
Immunofluorescence staining of CatL1 proteases of adult *F*. *gigantica* using the specific MoAb 4E3 as a probe. (A) The negative control of a cross section stained with myeloma culture fluid, showing tegument (Te), spine (Sp), muscle (Mu), caecum (Ca), and parenchymal cells (Pc), while vitelline glands (Vi) appear only nonspecific orange autofluorescence. (B) A low magnification micrograph showing intense fluorescence in both caecal epithelium and in the lumen of the caecum, while the tegument (Te), spine (Sp), muscle (Mu) and parenchymal cells (Pc) are not stained. (C and D) Medium and high magnification micrographs showing intense fluorescence in both caecal epithelium and in the lumen of the caecum, while parenchymal cells (Pc) are not stained.

The distribution of CatL1 antigen in adult *F*. *gigantica* was examined by indirect immunofluorescence staining using the MoAb 4E3. The positions and intensities of greenish fluorescence indicated the location and relative concentration of CatL1 that were bound to the MoAb. The myeloma culture fluid was used as a negative control and exhibited no staining in any tissues of the parasite, while vitelline glands showed only nonspecific orange autofluorescence (Fig 2A). When the parasite cross sections were treated with the MoAb, the fluorescence was very intense in both caecal epithelium and the caecal lumen, whilst the tegument, spine, muscle and parenchymal cells were not stained (Fig 2B–2D).

### The lower detection limits and the specificities of sandwich ELISA and IC strip test

**Fig 3:**
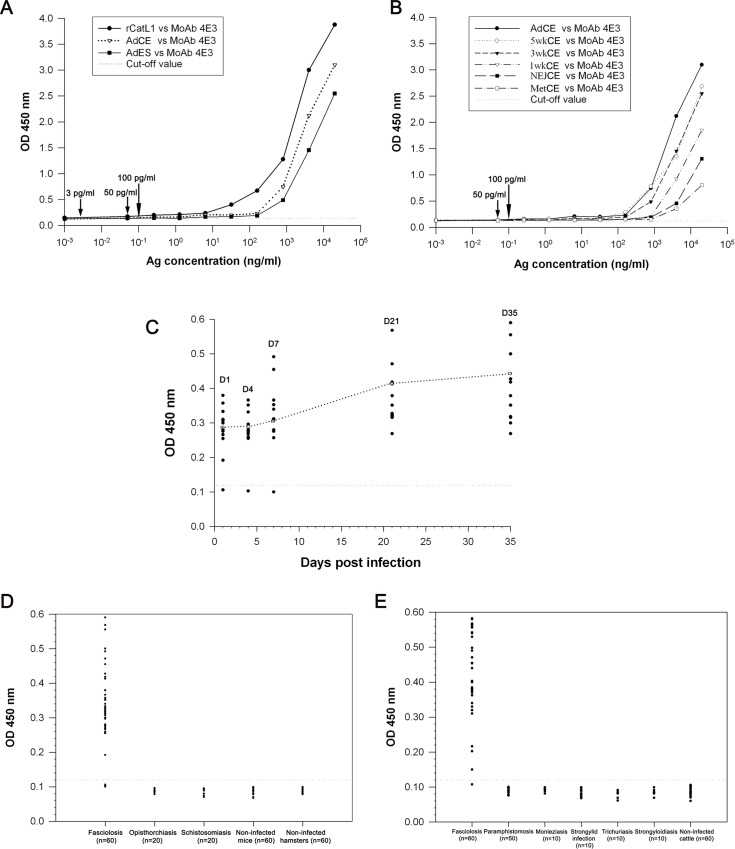
A reliable monoclonal antibody (MoAb)-based sandwich enzyme-linked immunosorbent assay (sandwich ELISA) is developed for diagnosis of fasciolosis by *F*. *gigantica*. (A and B) The lowest concentrations of rCatL1, CatL1 in CE of variuos stages of *F*. *gigantica*, and in ES antigens of adult *F*. *gigantica* as detected by the sandwich ELISA. (A) Lines with black circle, white triangle and black square denote the concentration levels of rCatL1 and CatL1 in CE and ES antigens of adult *F*. *gigantica*. The arrows indicate the lowest concentrations of CatL1 that could still be detected. (B) Lines with black circle, white circle, black triangle, white triangle, black square and white square show the concentration levels of CatL1 in CE of adult, 5-, 3-, 1-week-old juveniles, NEJ and Met of *F*. *gigantica*, respectively. The arrows indicate the lowest concentrations that CatL1 could still be detected. (C) Detection of circulating CatL1 antigens in the serum samples of mice experimentally infected with *F*. *gigantica* as compared with non-infected mice by sandwich ELISA. Black circles denote OD values of individual mouse serum; white squares represent mean OD values of all mice in each experimental group. The horizontal dotted line represents the cut-off value for a positive detection. (D) The relative levels of circulating CatL1 antigens in the serum samples from mice infected with *F*. *gigantica* and *S*. *mansoni* as well as hamster infected with *O*. *viverrini* as examined by sandwich ELISA (OD values at 450 nm). The serum samples from non-infected mice and hamsters are used as negative controls. The horizontal dotted line is the cut-off value for a positive detection. (E) The relative levels of circulating CatL1 antigens in the serum samples from cattle naturally infected with *F*. *gigantica* (fasciolosis), *P*. *cervi* (paramphistomosis), *M*. *benedeni* (Monieziasis), Strongylids (strongylid infection), *Trichuris* sp. (Trichuriasis), and *Strongyloides* sp.(strongyloidiasis) as measured by sandwich ELISA (OD values at 450 nm). Serum samples from non-infected cattle are used as negative controls. The horizontal dotted line represents the cut-off value for a positive detection.

For sandwich ELISA, the cut-off value was 0.124 and the lower detection limit of this assay system was determined using serial concentrations of rCatL1, CE and ES antigens of adult *F*. *gigantica*. Based on the lowest concentrations of antigen that still showed the sensitivity of test, this assay could detect rCatL1 and CatL1 in CE and ES fractions of *F*. *gigantica* at the lowest concentrations of 3, 50 and 100 pg/ml, respectively (Fig 3A). In addition, the lowest concentration for CE in Met, NEJ, 1-week-old juvenile antigens was at 100 pg/ml, and for 3-, 5-week-old juveniles and adult antigens was at 50 pg/ml (Fig 3B). Likewise, this test was highly specific for CatL1, as no cross-reactivity was shown when the assay was used to detect this antigen at various concentrations of other parasite antigens. The OD values established with *F*. *gigantica* were significantly higher than those developed with a panel of other parasite antigens (for example, more than 3.0 for *F*. *gigantica* compared with less than 0.1 for other species), as shown in Table 1.

**Table 1:** Specificity testing of a sandwich ELISA to various crude preparations from trematode, cestode, and nematode parasite antigens.

Antigen^a^	OD at 450 nm^b^
*F*. *gigantica*, rCatL1	3.961
*F*. *gigantica*	3.281
*F*. *hepatica*	0.091
*G*. *explanatum*	0.080
*E*. *pancreaticum*	0.081
*P*. *cervi*	0.087
*P*. *gracile*	0.086
*C*. *cotylophorum*	0.072
*F*. *cobboldi*	0.084
*G*. *crumenifer*	0.083
*S*. *spindale*	0.081
*M*. *benedeni*	0.066
*A*. *centripunctata*	0.062
*Trichuris* sp.	0.080
*H*. *placei*	0.076
*S*. *labiato-papillosa*	0.067
*S*. *mansoni*	0.081
*S*. *mekongi*	0.086
*S*. *japonicum*	0.085
*O*. *viverrini*	0.076
Reagent blank	0.151

^a^ The protein content of each parasite antigen preparation was adjusted to 20 μg/ml, and a 50-μl volume was used for analysis.

^b^ Mean OD was determined in triplicates performed on three separate occasions.

**Fig 4:**
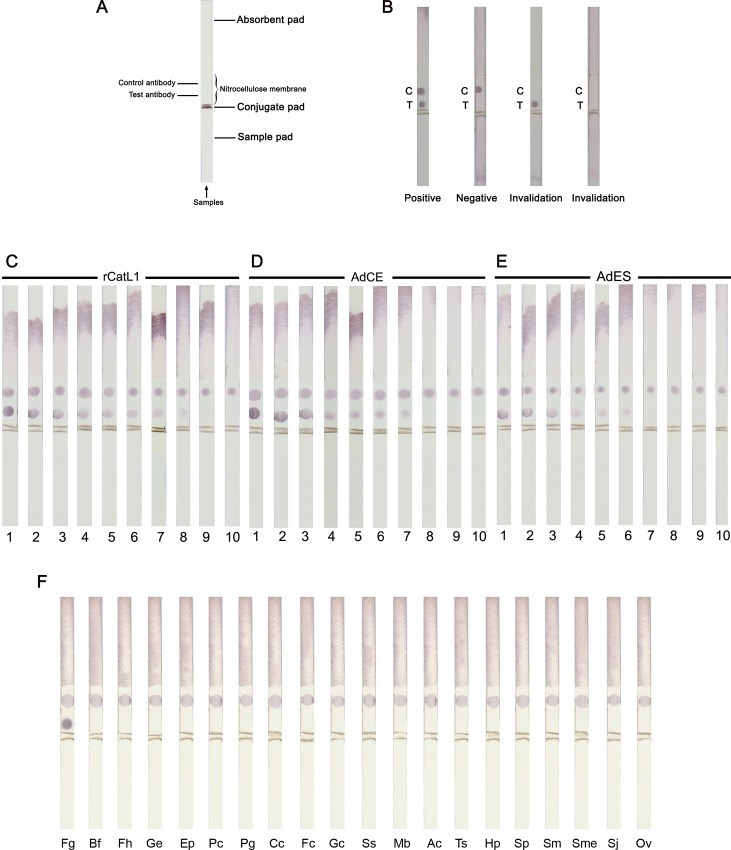
An immunochromatographic (IC) strip test is developed for diagnosis of fasciolosis by *F*. *gigantica*: Experiment trial. (A) A schematic diagram of the immunochromatographic (IC) strip test showing several components: a sample pad, a conjugate pad, an immobilized nitrocellulose membrane (control and test antibody) and an absorbent pad. (B) The samples of the IC strip test for deciding the results: a positive result shows two red dots at the test and control regions, while a negative result exhibits only one red dot in the control region. The strip tests are invalid when there is no red dot at the control region. (C-E) Sensitivity testing of the IC strip was studied using a series of dilutions in a buffer (0.0512–20,000 ng/ml) of adult *F*. *gigantica* recombinant cathepsin L1 (rCatL1) (C), crude extract (CE) (D), and excretory-secretory (ES) antigens (E). 1 = 20,000 ng/ml, 2 = 4,000 ng/ml, 3 = 800 ng/ml, 4 = 160 ng/ml, 5 = 32 ng/ml, 6 = 6.4 ng/ml, 7 = 1.2 ng/ml, 8 = 0.256 ng/ml, 9 = 0.0512 ng/ml, and 10 = buffer control. (F) The specificity of the IC strips were tested against CE from *F*. *gigantica*, other trematode, cestode and nematode parasites. All antigens were tested at 1,000 μg/ml. Fg = *F*. *gigantica*, Bf = buffer control, Fh = *F*. *hepatica*, Ge = *G*. *explanatum*, Ep = *E*. *pancreaticum*, Pc = *P*. *cervi*, Pg = *P*. *gracile*, Cc = *C*. *cotylophorum*, Fc = *F*. *cobboldi*, Gc = *G*. *crumenifer*, Ss = *S*. *spindale*, Mb = *M*. *benedeni*, Ac = *A*. *centripunctata*, Ts = *Trichuris* sp., Hp = *H*. *placei*, Sp = *S*. *labiato-papillosa*, Sm = *S*. *mansoni*, Sme = *S*. *mekongi*, Sj = *S*. *japonicum* and Ov = *O*. *viverrini*.

For the IC strip assay, the shematic details of the strip components are shown in Fig 4A, and the judgements of the results detected by strip test are also shown in Fig 4B. The lower detection limit or sensitivity of this assay was investigated by testing samples containing serial concentrations of rCatL1, CE and ES antigens of *F*. *gigantica*. The results revealed that the assay could detect rCatL1 and CatL1 in CE and ES fractions as low as 0.256, 1.2 and 6.4 ng/ml, respectively (Fig 4C–4E). Similar to sandwich ELISA, IC strip test showed high specificity for *F*. *gigantica* CatL1 antigen, and the cross-reactions with other parasite antigens were not observed. Moreover, the specificity ratio of this test is more than 833 times when all other antigens were tested at 1,000 μg/ml (Fig 4F). Furthermore, the IC strips were stable for at least 8 months, which the test still gave the same results.

### Detection of circulating CatL1 antigen in sera from infected mice and cattle by sandwich ELISA and IC strip test

#### Experimentally infected mice

**Fig 5:**
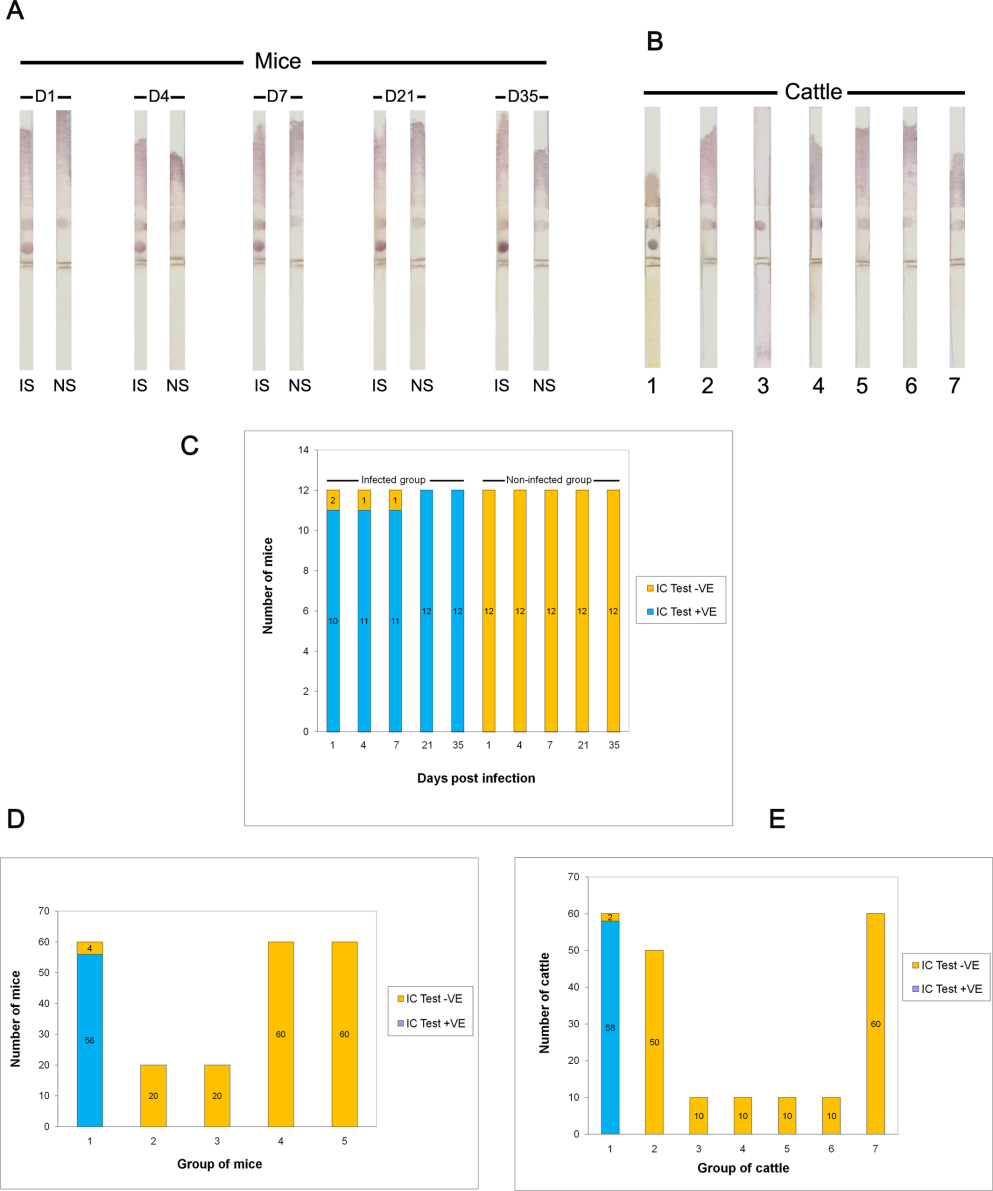
A field trial of the immunochromatographic (IC) strip test for diagnosis of fasciolosis by *F*. *gigantica*. (A) Detection of circulating CatL1 antigens in the serum samples of mice experimentally infected with *F*. *gigantica* as compared with non-infected mice. D1 = day 1 post infection, D4 = day 4 post infection, D7 = day 7 post infection, D = day 21 post infection, D35 = day 35 post infection, IS = infected serum, and NS = normal serum. (B) Detection of circulating CatL1 antigens in the serum samples of cattle naturally infected with *F*. *gigantica* (strip 1), *P*. *cervi* (strip 2), *M*. *benedeni* (strip 3), Strongylids (strip 4), *Trichuris* sp. (strip 5), and *Strongyloides* sp. (strip 6). Serum sample from non-infected cattle was used as the negative control (strip 7). (C) Detection of circulating CatL1 antigens in the serum samples of mice experimentally infected with *F*. *gigantica* as compared with non-infected mice, showing results of positivity (+VE) and negativity (-VE) as measured by IC test. (D and E) Comparison between positivity (+VE) and negativity (-VE) of different parasite species as measured by IC test. (D) Sera from mice experimentally infected with fasciolosis (1), opisthorchiasis (2), schistosomiasis (3), non-infected mice (4), and non-infected hamsters (5). (E) Sera from cattle naturally infected with fasciolosis (1), paramphistomosis (2), monieziasis (3), strongylid infection (4), trichuriasis (5), strongyloidiasis (6) and non-infected cattle (7).

The sandwich ELISA showed the number of infected mice sera and the number of positive sera as well as the percentages of positive sera at day 1, 4, 7, 21 and 35 post infection were 91.67% (11 out of 12), 91.67% (11 out of 12), 91.67% (11 out of 12), 100% (12 out of 12) and 100% (12 out of 12), respectively. The mean ODs of infected mice sera were significantly different from those of non-infected sera at day 1, 4, 7 (p < 0.05), 21 and 35 (p < 0.01) post infection. The antigen in infected sera began to be detectable at day 1 post infection (Fig 3C). As shown in Fig 5A and 5C, CatL1 antigen was also detected at days 1 to 35 post infection by the IC strip test in 56 of the 60 serum samples of mice infected with fasciolosis.

In addition, serum samples collected from 60 fasciolosis mice, 20 schistosomiasis mice, 20 opisthorchiasis hamsters as well as 60 and 60 non-infected mice and hamsters were tested. It was found that 95% (57/60) (Fig 3D) and 93% (56/60) (Fig 5D) fasciolosis sera were tested positive with sandwich ELISA and IC test, respectively, while all 100% (160/160) sera from those with other parasitic infections and normal control animals were negative. Hence, the diagnostic sensitivity, specificity, positive predictive value, negative predictive value, false positive rate, false negative rate and accuracy were calculated as 95%, 100%, 100%, 98.2%, 0%, 5% and 98.6% for sandwich ELISA, as well as 93%, 100%, 100%, 97.6%, 0%, 6.7% and 98.2% for IC test (Table 2).

**Table 2:** A comparison of diagnostic values of the sandwich ELISA and IC test for CatL1 antigen detection in sera of mice experimentally and cattle naturally infected with *F*. *gigantica*.

Parameters	Experimentally infected mice (%)		Naturally infected cattle (%)	
	Sandwich ELISA	IC test	Sandwich ELISA	IC test
1. Sensitivity	95	93	98.3	96.7
2. Specificity	100	100	100	100
3.Positive predictive value	100	100	100	100
4. Negative predictive value	98.2	97.6	99.3	98.7
5. False positive rate	0	0	0	0
6. False negative rate	5	6.7	1.7	3.3
7. Accuracy	98.6	98.2	99.5	99.1

#### Naturally infected cattle

All serum samples from the 210 cattle were collected and examined by sandwich ELISA and IC test as follows: 60 sera from fasciolosis cattle, 50 from paramphistomosis cattle, 10 from monieziasis cattle, 10 from strongylid infections cattle, 10 from trichuriasis cattle, 10 from strongyloidiasis cattle and 60 from non-infected cattle.

The results revealed that 98.3% (59/60) (Fig 3E) and 96.7% (58/60) (Fig 5B and 5E) of fasciolosis sera were tested positive by sandwich ELISA and IC test, respectively, whilst all 100% (150/150) sera from those infected with other infections and non-infected controls were found to be negative. Therefore, the diagnostic sensitivity, specificity, positive predictive value, negative predictive value, false positive rate, false negative rate and accuracy were found to be 98.3%, 100%, 100%, 99.3%, 0%, 1.7% and 99.5% for sandwich ELISA, and be 96.7%, 100%, 100%, 98.7%, 0%, 3.3% and 99.1% for IC test, as shown in Table 2.

### Indirect ELISA for the detection of antibody against CatL1 antigen of *F*. *gigantica*

For the antibody detection by indirect ELISA, the numbers of sera from infected mice being tested and percentages of positive detection during the period of 1–5 weeks post infection were 8.33% (1 out of 12) at day 1, 25% (3 out of 12) at day 4, 25% (3 out of 12) at day 7, 50% (6 out of 12) at day 21, and the percent reached the highest level at day 35 (75%) (9 out of 12). The mean OD value for mouse antibody diagnosed as having fasciolosis at day 35 post infection is significantly different from that of the control sera OD (P < 0.05).

## Discussion

Up to now, the available methods for diagnosis of fasciolosis in ruminants were microscopic detection of eggs in the feces or antibody detection. The results from both tests do not reflect the parasite burden at the time of detection because eggs appear in the host’s feces quite late at approximately at 16 weeks post infection when the parasites reach adult stage, while the antibody may persist even after the parasites disappear or being killed. In order to achive this, it was necessary to find the more accurate and convenient diagnostic assays. In the present study, we developed a MoAb-based sandwich ELISA for detection of circulating CatL1 antigen in the serum samples from mice experimentally and cattle naturally infected with *F*. *gigantica*. In addition, we were the first to develope the immunochromatographic (IC) strip test for rapid detection of fasciolosis by *F*. *gigantica* infection. The MoAb 4E3 that we produced is quite specific to FgCatL1 and showed no cross-reactions with antigens of other parasites, including *F*. *hepatica*, *G*. *explanatum*, *E*. *pancreaticum*, *P*. *cervi*, *P*. *gracile*, *C*. *cotylophorum*, *F*. *cobboldi*, *G*. *crumenifer*, *S*. *spindale*, *M*. *benedeni*, *A*. *centripunctata*, *Trichuris* sp., *H*. *placei*, *S*. *labiato-papillosa*, *S*. *mansoni*, *S*. *mekongi*, *S*. *japonicum and O*. *viverrini*. This suggested that this MoAb might binds to a specific epitope, which is only present in *F*. *gigantica* CatL1. Also, the immunolocalization study demonstrated that this MoAb could detect the native CatL1 in caecal epithelium and lumen of adult *F*. *gigantica* with the same result of that reported by Meemon et al. [25] using a mouse polyclonal antibody against CatL of adult *F*. *gigantca*. This confirms the high specificity of the MoAb to CatL1. Therefore, we could safely say that this MoAb 4E3 can detect the circulating CatL1 antigen in both early and late stages of infection.

In our previous study, we successfully produced MoAb against the 28.5 kDa tegumental antigen (TA) of adult *F*. *gigantica* for the detection of this circulating antigen in the sera of mice experimentally infected with fasciolosis using a sandwich ELISA [12]. Recently, we also produced a MoAb 2F9 specific to rCatB3 of *F*. *gigantica* and developed a reliable MoAb-based sandwich ELISA for detection of circulating CatB3 in experimentally infected mice and naturally infected cattle sera. It was found that these circulating antigens could be detected as early as day 1 to day 35 after infection [13]. In addition, Viyanant et al., [10] reported that a MoAb-based sandwich ELISA was used to detect circulating 66 kDa TA in the serum samples of cattle experimentally and naturally infected with *F*. *gigantica*. However, this MoAb showed cross-reaction with antigens from other trematode parasites and the MoAb was unstable. Similarly, Fagbemi et al. [32] used MoAb to detect whole worm antigens in cattle infected with *F*. *gigantica*; however, the MoAb showed strong cross-reactions with other parasite antigens. In addition, Wongwairot et al. [33] revealed that MoAb could detect isoforms of cathepsin Ls of both juvenile and adult *F*. *gigantica*. Another study of *F*. *gigantica* reported that the diagnosis of human fasciolosis was developed by using MoAb against 49.5 kDa ES antigens of *F*. *gigantica*, but the MoAb also exhibited cross-reactivity with *Schistosoma mansoni* antigen [34].

In the present study, our MoAb 4E3 was able to detect rCatL1 and CatL1 antigens in CE and ES fractions of adult *F*. *gigantica* at the concentrations as low as 3, 50 and 100 pg/ml, respectively. In addition, the CatL1 antigen could also be detected in CE of 3-, 5-week-old juvenile and adult *F*. *gigantica* at a minimum of 50 pg/ml and in Met, NEJ and 1-week-old juvenile *F*. *gigantica* at 100 pg/ml. This could be attributed to the use of the rabbit anti-mouse IgG as the precoating antibody which helped to increase the amount of MoAb on the well surface. Moreover, the use of MoAb as the capture antibody enhanced the specific binding that helped to reduce the background and allow a very low detection limit of this assay. The detection limits of our assay are lower than those reported earlier; for example, Langley and Hillyer [8] reported that their sandwich ELISA could detect the circulating ES antigens in the sera of mice infected with *F*. *hepatica* at low detection limit of 0.25 ng/ml. As well, our assay’s detection limits are lower than that of Espino and Finlay [35] who used a sandwich ELISA for detection of *F*. *hepatica* ES antigens in patients’ stool samples and found the detection limit was at 15 ng/ml. In addition, the MoAb (MM3) capture ELISA was developed for the detection of ES antigens in fecal samples of animals infected with *F*. *hepatica* with the detection limits at 0.3 (in sheep) and 0.6 ng/ml (in cattle) [36]. Our earlier study, we developed a MoAb-based sandwich ELISA for the detection of circulating *F*. *gigantica* 28.5 kDa TA in the sera of experimentally infected mice and found that the lower detection limit of this antigen in the TA, CE and ES antigens was 600 pg/ml, 16 ng/ml and 60 ng/ml, respectively, which were considerably higher than in the present study [12]. Furthermore, we found that a sandwich ELISA detecting circulating cathepsin B3 (CatB3) antigen in the serum samples of mice and cattle infected with *F*. *gigantica* with the lower detection limit at 10, 100 and 400 pg/ml, when appiled for the detection of rCatB3 antigen and CatB3 in CE of Met and NEJ of *F*. *gigantica*, respectively, which were still higher than in this study [13]. Likewise, the lower detection limits reported herein are lower than that reported by Demerdash et al. [37] who used a sandwich ELISA to detect the ES antigens in both serum and stool samples of patients infected with *F*. *gigantica* at a concentration level 3 ng/ml. In this study, we found that the diagnostic sensitivity, specificity and accuracy of this MoAb-based sandwich ELISA were very high as in experimentally infected mouse sera at 95%, 100% and 98.6%, whereas in naturally infected cattle sera they were 98.3%, 100% and 99.5%, respectively. Our results indicated that this sandwich ELISA could be successfully applied to naturally infected cattle with a large body size.

For the time on antigen-detecion assays, Langley and Hillyer [8] reported that circulating antigen was detected in the sera of mice infected with *F*. *hepatica* at the 1st week of infection using rabbit antiserum. Likewise, the circulating 66 kDa antigen of *F*. *gigantica* was detected in the sera of experimentally infected cattle at the first week post infection [10]. Fagbemi et al. [9] could detect circulating 88 kDa antigen in the sera of cattle experimentally infected with *F*. *gigantica* during the 2nd and 3rd weeks after infection. In addition, Velusamy et al. [11] detected the circulating 54 kDa antigen of *F*. *gigantica* in the sera of experimentally infected cattle at the 2nd week post infection. On the other hand, our assay could detect the circulating CatL1 antigen in the serum samples of mice experimentally infected with *F*. *gigantica* as early as the 1st day of infection, which is much earlier than in previous reports. This data herein is similar to the results of our previous studies [12–13]. In our assay, the OD value of antigen level increased significantly from day 1 to day 7 post infection, and increased steadily to day 21 post infection; then it became stable from days 21 to day 35 (Fig 3C). This probably reflects the slow and continuous release of this antigen into the hosts’ circulation during the first few weeks after infection. Once the parasites reached mature stage in the bile ducts, a large amount of antigens were released into the hosts’ circulation.

Recently, immunochromatographic (IC) strip assay has been developed for the rapid detection of an analyte. This test is based on a simplified version of ELISA and easy to perform and do not require any equipment nor expertise. IC test shows the results with a few minutes and therefore is very useful in physicians’ office where immediate test results could be obtained. It was reported that IC test has been widely used in the medical fields, i.e., the detection of biological infectious agents and chemical contaminants such as bateria, viruses, toxins as well as drugs and pesticides [38–40], but rarely applied in veterinary fields. Up to now, there is no report on the detection of circulating *F*. *gigantica* antigens by IC assay. Hence, this research is the first study that described the IC test for the rapid detection of fasciolosis by *F*. *gigantica*.

In the present study, IC results exhibited very high sensitivity and specificity for the detection of circulating CatL1 antigen in both mice experimentally and cattle naturally infected with *F*. *gigantica*. It could detect rCatL1 and CatL1 antigens in CE and ES fractions of adult *F*. *gigantica* at the concentration as low as 0.256, 1.2 and 6.4 ng/ml, respectively. More interestingly, no cross-reaction was observed when the IC strip was employed to detect the similar antigens from other trematode, cestode and nematode parasites. The sensitivity, specificity and accuracy of this assay were very high as in the sera of experimentally infected mice these values were 93%, 100% and 98.2%, while for naturally infected cattle they were 96.7%, 100% and 99.1%, respectively. Similar to sandwich ELISA assay, this finding indicated the advantage of this test as a suitable diagnostic tool for the detection of pre-patent and current infection. It was possible to detect circulating antigen as early as first week post infection in experimentally infected mice sera. Hence, this assay is a simple, fast and could be useful in laboratories as well as veterinary fields lacking well-trained parasitologists.

## Conclusions

From our finding, both sandwich ELISA and IC strip tests exhibited high efficiencies and precisions. These two assay methods could be used as the important diagnostic tools not only for the rapid detection of fasciolosis but also for the seroepidemiological screening of cattle from different regions, which in turn could contribute to the monitoring and control of the disease.
